# 76例头颈部腺样囊性癌伴肺转移患者的生存分析

**DOI:** 10.3779/j.issn.1009-3419.2021.102.21

**Published:** 2021-06-20

**Authors:** 振 余, 磊 于, 晓红 陈, 兴国 杨, 葆勋 张, 涛 于, 鑫 杜

**Affiliations:** 1 100730 北京，首都医科大学附属北京同仁医院胸外科 Department of Thoracic Surgery, Beijing Tongren Hospital, Capital Medical University, Beijing 100730, China; 2 100730 北京，首都医科大学附属北京同仁医院耳鼻咽喉头颈外科 Department of Otolaryngology Head and Neck Surgery, Beijing Tongren Hospital, Capital Medical University, Beijing 100730, China

**Keywords:** 腺样囊性癌, 头颈部肿瘤, 肺转移, Adenoid cystic carcinoma, Head and neck neoplasms, Lung metastasis

## Abstract

**背景与目的:**

头颈部腺样囊性癌（adenoid cystic carcinoma, ACC）常发生肺转移，目前ACC肺转移的研究报道不多，对于其确切的临床特征和治疗结果知之甚少，最佳治疗策略尚无共识。本研究探讨头颈部ACC肺转移的有效治疗策略、临床结局和长期预后。

**方法:**

回顾性分析76例头颈部ACC肺转移患者的临床和随访资料。根据初始治疗分为手术、手术+放化疗、放化疗、支持治疗四组；根据国际肺转移分期系统（International Registry of Lung Metastases Staging System, IRLM）进行分期。采用*Kaplan-Meier*法、*Log-rank*检验比较不同治疗方式及不同IRLM分期患者的总生存期（overall survival, OS）及无进展生存期（progression-free survival, PFS）的统计学差异。

**结果:**

接受手术治疗患者的OS及PFS优于支持性治疗或放疗/化疗的患者（OS: *P* < 0.000, 1; PFS: *P* < 0.000, 1）；IRLM低分期的OS和PFS优于高分期患者（OS: *P* < 0.000, 1; PFS: *P* < 0.000, 1）；单发肺转移、不合并胸腔积液患者具有更好的OS和PFS。

**结论:**

接受手术治疗的ACC肺转移患者的远期预后与更好的OS和PFS相关。对于具备手术条件的ACC肺转移的患者，进行完整手术切除的意义高于其他治疗方案。

## Abstract (en)

**Background and objective:**

Adenoid cystic carcinoma (ACC) of the head and neck often develops lung metastasis. At present, there are not many research reports on ACC lung metastasis, little is known about its exact clinical features and treatment results, and there is no consensus on the best treatment strategy. This study explored the effective treatment strategies, clinical outcomes and long-term prognosis of head and neck ACC lung metastases.

**Methods:**

The clinical and follow-up data of 76 patients with head and neck ACC lung metastases were retrospectively analyzed. According to the initial treatment of patients, they are divided into 4 groups: surgery, surgery+chemotherapy or radiotherapy, chemotherapy or radiotherapy and supportive treatment. The patients were staged according to the International Registry of Lung Metastases Staging System (IRLM). *Kaplan-Meier* method and *Log-rank* test were used to compare the statistical differences of overall survival (OS) and progression-free survival (PFS) of patients with different treatment methods and different IRLM stages.

**Results:**

The OS and PFS of patients undergoing surgery are better than those of supportive therapy or radiotherapy and/or chemotherapy (OS: *P* < 0.000, 1; PFS: *P* < 0.000, 1). The OS and PFS of patients with low stage IRLM are better than those with high stage (OS: *P* < 0.000, 1; PFS: *P* < 0.000, 1). Patients with single lung metastasis and without pleural effusion have better OS and PFS.

**Conclusion:**

The long-term prognosis of patients with lung metastasis of head and neck ACC who undergo surgery is better than other treatments, which is related to higher OS and PFS. For patients with ACC lung metastases who are operationally eligible, the significance of complete surgical resection should be higher than other treatment options.

腺样囊性癌（adenoid cystic carcinoma, ACC）是一种较少见的恶性肿瘤，发病率为百万分之四，起源于外分泌腺，尤其是大小涎腺，约占头颈部恶性肿瘤的1%^[1]^。虽然ACC的临床进程相对缓慢，很少与局部淋巴结受累相关，但25%-50%的ACC患者会在病程中发生迟发性血行转移，这种转移最常见于肺部^[2, 3]^。有报道^[1, 4]^称，ACC患者长期生存率低主要与未能控制远处转移有关。只有少数报告^[2, 4, 5]^描述了ACC肺转移病灶切除的临床结果，对于其确切的临床、病理学特征和治疗结果知之甚少，最佳治疗策略尚无共识^[6-8]^，而且，目前并不清楚ACC肺转移患者是否都能从特异性治疗（如手术或放化疗）中获益^[1, 9, 10]^。本研究旨在探讨ACC肺转移患者的有效治疗方式、临床结局和长期预后。

## 1 资料与方法

### 1.1 一般资料

回顾性选择2010年1月-2019年12月就诊于首都医科大学附属北京同仁医院并明确诊断为头颈部ACC且伴有肺转移的患者共87例。所有患者在研究入组时原发病灶经过治疗已得到控制，均无除肺转移以外的其他脏器的远处转移；排除合并其他原发性恶性肿瘤病史的患者。87例患者中，7例患者因病历资料信息不完整而被排除，4例因5年内合并其他恶性肿瘤而被排除。最终有76例ACC肺转移患者被纳入研究，患者的临床及病理资料均从本机构前瞻性数据库中获取。首都医科大学附属北京同仁医院伦理评审委员会批准了这项研究。

### 1.2 检查及治疗方式

常规的治疗前评估检查项目包括胸部、颈部计算机断层扫描（computed tomography, CT）、腹部超声、脑磁共振成像（magnetic resonance imaging, MRI）、核医学骨扫描/18-氟脱氧葡萄糖正电子发射断层扫描（positron emission tomography-CT, PET-CT）。因考虑费用方面的问题，PET-CT并非强制性要求，与其他检查结合使用。病理专科医师重新检查了准备好的载玻片，确认没有病例需要修改诊断。研究期间，采用多学科方法建立治疗模式。简言之，病灶可完全切除时优先考虑手术治疗。手术方式依据肺转移瘤部位，视具体情况行亚肺叶或肺叶切除术。术后根据病理分期确定辅助治疗。如果由于中央气道病变或胸膜转移，或患者自身状态或意愿不适合手术，则对患者进行化疗（以铂类为基础的双药或三药方案^[11]^，本研究中接受“环磷酰胺+表柔比星+顺铂”方案化疗13例，“吉西他滨+顺铂”方案化疗14例，“环磷酰胺+阿霉素+顺铂”方案化疗3例）或局部放疗（6周内接受40 Gy-60 Gy放射剂量^[4, 12]^，本研究中接受40 Gy放疗5例，接受46 Gy放疗11例，接受52 Gy放疗4例，接受60 Gy放疗1例）或辅助支持治疗。按患者初始治疗方法分为4组：单纯手术组被定义为接受手术切除的患者；手术联合放疗或化疗组被定义为接受手术切除联合放疗或化疗的患者；放疗或化疗组被定义为接受放疗或化疗的患者；辅助支持治疗组被定义为因任何原因未行手术或放化疗干预、仅接受其他辅助支持治疗的患者。

### 1.3 数据收集

回顾性分析76例患者的人口统计学特征、原发肿瘤部位、组织学类型、诊断时的临床分期、首次治疗方式等。本研究主要评估ACC肺转移病变接受治疗后的相关结局及预后，我们考虑了无病间期（disease-free interval, DFI）、国际肺转移分期、肺转移瘤大小、肺转移瘤数量、是否合并胸腔积液和手术切除的完整性，并评估了它们与总生存期（overall survival, OS）和无进展生存期（progression-free survival, PFS）的关系。DFI定义为原发肿瘤初次治疗至肺转移发生的时间间隔。本研究根据国际肺转移分期系统（International Registry of Lung Metastases Staging System, IRLM）^[1]^将ACC肺转移患者分为4期：Ⅰ期（手术完整切除，DFI≥36个月和单处转移）；Ⅱ期（手术完整切除，DFI < 36个月或多发转移）；Ⅲ期（手术完整切除，DFI < 36个月和多发转移）；Ⅳ期（手术无法完整切除）。使用美国麻醉医师协会（American Society of Anesthesiologists, ASA）的身体状况分类对接受手术的患者的心肺功能进行评估，不良表现状态被定义为ASA Ⅲ级和Ⅳ级。

### 1.4 随访

术后1个月及术后每6个月随访进行胸部CT检查。随访时间开始于初次接受治疗日期，结束于死亡或2019年12月31日（以最先发生者为准）。在门诊复查和电话访谈中检查患者的生存状态。最后一次检查后至少1年没有电话联系的患者被认为失去随访。从第一次ACC肺部转移病变接受治疗到死亡或最后一次随访（删失）计算OS。从第一次肺部病变接受治疗到肿瘤进展或最后一次随访（删失）计算PFS。

### 1.5 统计学方法

使用R（version 3.4.3）软件进行统计学分析。连续变量的数据以均数±标准差（Mean±SD）或中位数表示，计数资料以例数和百分率（%）表示。组间连续变量比较采用*t*检验或*Wilcoxon*秩和检验，分类资料比较采用*Fisher*精确检验或卡方检验。采用*Kaplan-Meier*法绘制生存曲线，*Log-rank*检验分析OS及PFS的统计学差异。通过生存曲线观察5年及10年生存率，累积生存率的计算是将各个时点的生存概率进行乘积计算而来，考虑了删失数据的影响。*P* < 0.05为差异具有统计学意义。

## 2 结果

### 2.1 基线资料及临床特点

本研究中，76例ACC肺转移患者中，35例（46.05%）为男性，41例（53.95%）为女性。中位年龄为49岁（范围为17岁-77岁）。原发肿瘤位于腮腺17例（22.37%）、颌下腺16例（21.05%）、舌下腺14例（18.42%）、上颌窦5例（6.58%）、咽部4例（5.26%）、鼻咽部6例（7.89%）、舌5例（6.58%）、气管6例（7.89%）。根据IRLM分期系统，Ⅰ期24例（31.58%），Ⅱ期26例（34.21%），Ⅲ期11例（14.47%），Ⅳ期15例（19.74%）。通过胸部CT/PET-CT扫描评估病灶的位置和数目，近一半患者表现为多发性肺转移（34例，44.74%）。61例患者（80.3%）接受了手术，每例患者切除的肺部病灶数目从1个到11个不等，其中32例（42.1%）接受了手术联合辅助放疗/化疗（表 1）。

**表 1:** 四组患者人口学特征及临床资料 Demographic characteristics and clinical data of four groups of patients

Characteristics	Surgery alone (*n*=29)	Surgery+CRT (*n*=32)	CRT (*n*=10)	Adjuvant therapy (*n*=5)	*P*
Age (yr)	43.83±17.23	48.03±15.88	50.40±0.83	58.20±6.72	0.233
Gender					0.217
Male	9 (31.03%)	17 (53.12%)	6 (60.00%)	3 (60.00%)	
Female	20 (68.97%)	15 (46.88%)	4 (40.00%)	2 (40.00%)	
BMI (kg/m^2^)	22.99±3.26	23.35±2.61	24.97±4.01	22.88±2.35	0.356
FEV_1_ (%)	87.83±6.25	87.90±6.51	82.17±8.31	86.58±7.98	0.115
Smoke					0.518
Never	25 (86.21%)	23 (71.88%)	9 (90.00%)	4 (80.00%)	
Current smokers	1 (3.45%)	5 (15.62%)	0 (0.00%)	1 (20.00%)	
Abstained for at least 1 year	3 (10.34%)	4 (12.50%)	1 (10.00%)	0 (0.00%)	
ASA grade					-
1	20 (68.97%)	23 (71.88%)	-	-	
2	7 (24.14%)	8 (25.00%)	-	-	
3	2 (6.90%)	1 (3.12%)	-	-	
Radiotherapy					< 0.001
No	29 (100.00%)	17 (53.12%)	4 (40.00%)	5 (100.00%)	
Yes	0 (0.00%)	15 (46.88%)	6 (60.00%)	0 (0.00%)	
Chemotherapy regimens					< 0.001
No	29 (100.00%)	7 (21.88%)	5 (50.00%)	5 (100.00%)	
Yes	0 (0.00%)	25(78.12%)	5 (50.00%)	0 (0.00%)	
Metastases largest diameter (cm)					0.435
< 2.5	20 (68.97%)	22 (68.75%)	8 (80.00%)	3 (60.00%)	
2.5-5.0	8 (27.59%)	6 (18.75%)	0 (0.00%)	1 (20.00%)	
> 5.0	1 (3.45%)	4 (12.50%)	2 (20.00%)	1 (20.00%)	
Number of metastatic tumors					0.395
Single	17 (58.62%)	19 (59.38%)	5 (50.00%)	1 (20.00%)	
Multiple	12 (41.38%)	13 (40.62%)	5 (50.00%)	4 (80.00%)	
Surgical procedures					< 0.001
None	0 (0.00%)	0 (0.00%)	10 (100.00%)	5 (100.00%)	
Wedge resection	12 (41.38%)	12 (37.50%)	0 (0.00%)	0 (0.00%)	
Lobectomy	8 (27.59%)	9 (28.12%)	0 (0.00%)	0 (0.00%)	
Segmentectomy	6 (20.69%)	10 (31.25%)	0 (0.00%)	0 (0.00%)	
Sleeve lobectomy	3 (10.34%)	1 (3.12%)	0 (0.00%)	0 (0.00%)	
IRLM					< 0.001
Ⅰ	10 (34.48%)	14 (43.75%)	0 (0.00%)	0 (0.00%)	
Ⅱ	15 (51.72%)	11 (34.38%)	0 (0.00%)	0 (0.00%)	
Ⅲ	4 (13.79%)	7 (21.88%)	0 (0.00%)	0 (0.00%)	
Ⅳ	0 (0.00%)	0 (0.00%)	10 (100.00%)	5 (100.00%)	
Combined with pleural effusion					< 0.001
None	26 (89.66%)	29 (90.62%)	8 (80.00%)	0 (0.00%)	
Unilateral pleural effusion	2 (6.90%)	2 (6.25%)	0 (0.00%)	4 (80.00%)	
Bilateral pleural effusion	1 (3.45%)	1 (3.12%)	2 (20.00%)	1 (20.00%)	
Categoric data are expressed as number (%) and continuous data as Mean±SD or median (interquartile range). BMI: body mass index; FEV_1_: forced expiratory volume in one second; ASA: American Society of Anesthesiologists; IRLM: International Registry of Lung Metastases Staging System.					

### 2.2 治疗策略与生存的关系

所有患者的中位随访时间为70个月（范围为33个月-126个月），中位OS为107个月，中位PFS为85个月。所有患者的5年OS率为76.67%（95%CI: 67.25%-87.42%），10年OS率为38.49%（95%CI: 25.35%-58.45%）。5年和10年PFS率分别为65.41%（95%CI: 55.21%-77.5%）和21.24%（95%CI: 10.66%-42.31%）。在所有治疗组中，手术切除联合放疗/化疗组的5年和10年OS率（87.92%, 71.05%）和PFS率（80.63%, 45.61%）最高（图 1A，图 1B），优于放化疗及辅助支持治疗的患者（OS: *P* < 0.000, 1; PFS: *P* < 0.000, 1），但与单纯手术组比较未见明显统计学差异（OS：*P*=0.084；PFS：*P*=0.34，图 2A，图 2B）。放疗及化疗组5年OS率和PFS率分别为40%（95%CI: 18.72%-85.45%）和30%（95%CI: 11.64%-77.32%），接受手术治疗患者的OS（*P* < 0.000, 1）及PFS（*P* < 0.000, 1）明显优于未接受手术行放化疗及辅助支持治疗的患者（图 2C，图 2D）。

**图 1:**
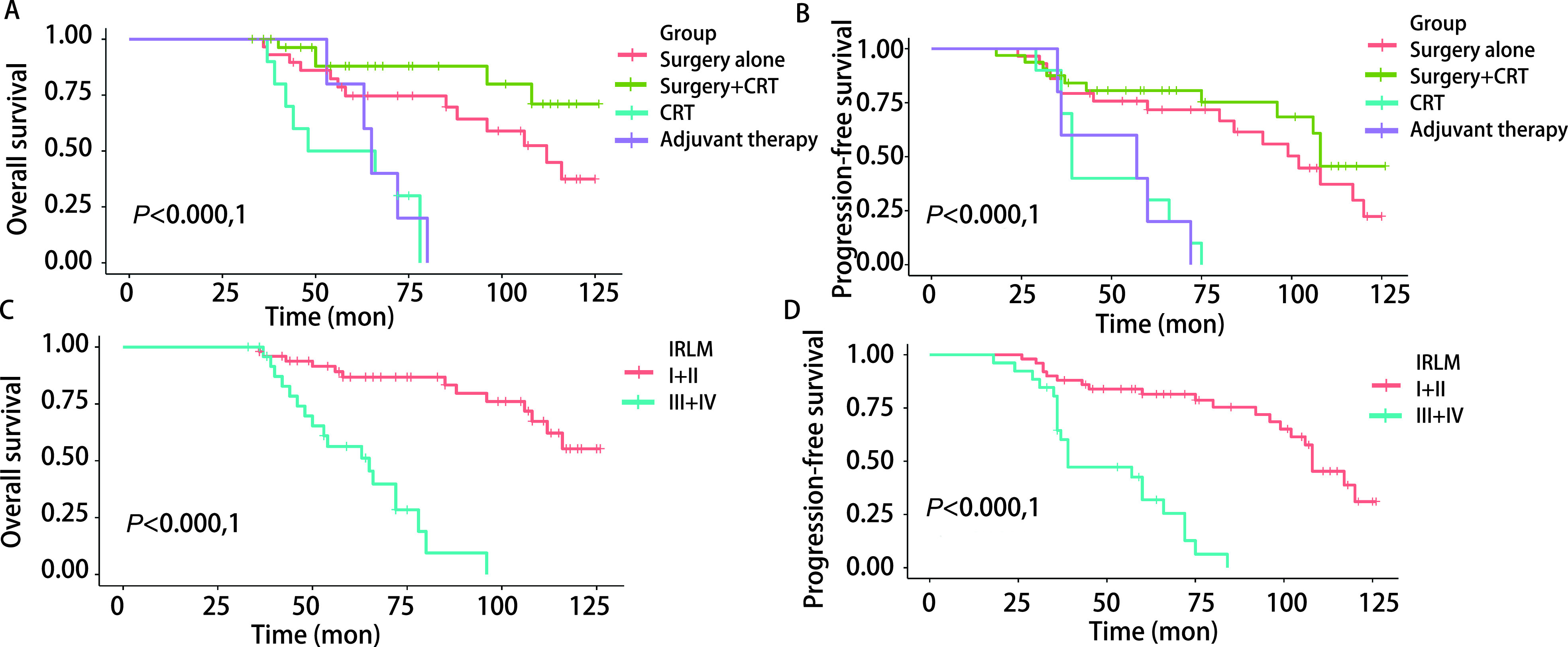
不同治疗组ACC肺转移患者OS（A）及PFS（B）生存曲线比较。按照IRLM分期系统，低分期（Ⅰ期+Ⅱ期）患者的OS（C）和PFS（D）明显优于高分期（Ⅲ期+Ⅳ期）患者。 Comparison of OS (A) and PFS (B) survival curves of patients with ACC lung metastases in different treatment groups. According to the IRLM, the OS (C) and PFS (D) of stage Ⅰ+Ⅱ patients are significantly better than those of stage Ⅲ+Ⅳ patients. PFS: progression-free survival.

**图 2:**
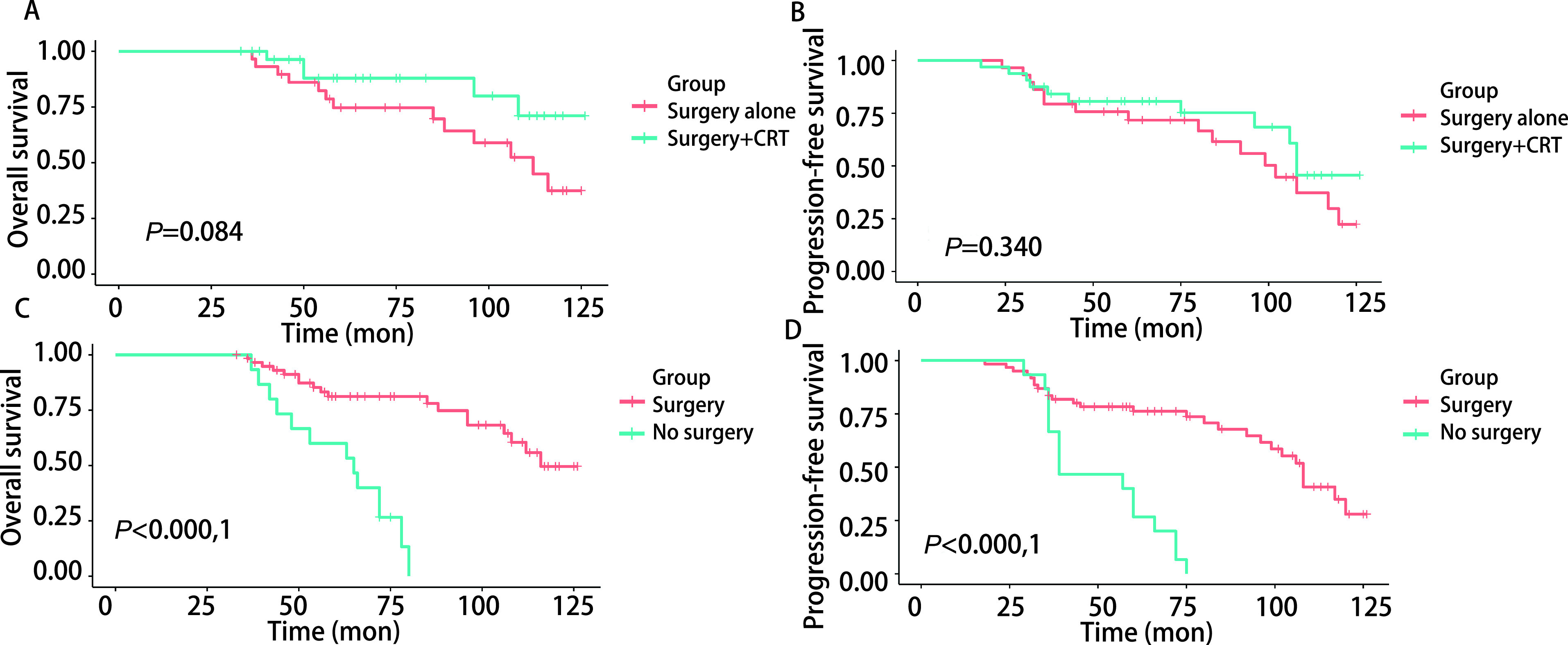
不同治疗方式ACC肺转移患者生存曲线比较。单纯手术组患者与手术切除联合放疗或化疗组患者的OS（A）及PFS（B）未见明显统计学差异；接受手术治疗的患者OS（C）及PFS（D）明显优于接受放化疗及辅助支持治疗的患者。 Comparison of survival curves of patients with ACC lung metastases with different treatments. There was no significant difference in OS (A) and PFS (B) between the surgery alone group and the surgery+CRT group; The OS (C) and PFS (D) of the patients in the surgery group were significantly better than those in the CRT or adjuvant therapy groups.

### 2.3 转移分期与生存的关系

按照IRLM分期系统，Ⅰ期和Ⅱ期ACC肺转移患者5年OS率为86.7%（95%CI: 77.42%-97.27%），10年OS率为55.26%（95%CI: 37.92%-80.54%），Ⅲ期和Ⅳ期患者5年和10年OS率分别为56.29%（95%CI: 39.18%-80.88%）和0%。DFI≥36个月与DFI < 36个月ACC肺转移患者5年OS率分别为91.04%（95%CI: 79.89%-100%）和67.14%（95%CI: 54.71%-82.38%），10年OS率分别为71.33%（95%CI: 52.25%-97.37%）和15.82%（95%CI: 5.07%-49.35%）。多因素分析结果显示，IRLM分期是ACC肺转移OS（Ⅰ+Ⅱ *vs* Ⅲ+Ⅳ: HR=7.93, 95%CI: 3.22-19.56, *P* < 0.000, 1）的独立危险因素（表 2）。ACC肺转移低分期（Ⅰ期+Ⅱ期）患者的OS（*P* < 0.000, 1；图 1C）和PFS（*P* < 0.000, 1；图 1D）明显优于高分期（Ⅲ期+Ⅳ期）患者。

**表 2:** ACC肺转移患者OS的单因素及多因素分析（*n*=76） Univariate and multivariate analysis of OS in patients with ACC pulmonary metastasis (*n*=76)

Variable	Univariate analysis				Multivariate analysis		
	HR	95%CⅠ	*P*		HR	95%CⅠ	*P*
Age	1.01	0.99-1.04	0.2350		1.01	0.98-1.03	0.580, 8
Gender							
Male	Reference				Reference		
Female	0.8	0.39-1.63	0.539, 9		0.67	0.31-1.43	0.299, 0
Smoke							
Never	Reference				Reference		
Current smokers	0.84	0.20-3.54	0.810, 3		0.61	0.13-2.81	0.526, 1
Abstained for at least 1 year	0.45	0.11-1.89	0.275, 0		0.37	0.09-1.61	0.184, 7
Treatment strategies							
Surgery alone	Reference				Reference		
Surgery+CRT	0.42	0.15-1.19	0.103, 6		0.37	0.12-1.13	0.080, 8
CRT	4.88	1.80-13.25	0.001, 9		4.56	1.47-14.13	0.008, 4
Adjuvant therapy	4.31	1.40-13.30	0.010, 8		3.03	0.76-12.09	0.115, 6
No. of metastasis							
Single	Reference				Reference		
Multiple	2.36	1.12-4.98	0.023, 9		3.10	1.38-6.97	0.006, 2
Combined with pleural effusion							
No	Reference				Reference		
Yes	5.18	2.34-11.49	< 0.000, 1		5.95	2.50-14.15	< 0.000, 1
IRLM							
Ⅰ+Ⅱ	Reference				Reference		
Ⅲ+Ⅳ	8.81	3.69-21.05	< 0.000, 1		7.93	3.22-19.56	< 0.000, 1
HR: hazard ratio; OS: overall survival.							

### 2.4 转移类型与生存的关系

本研究按肺转移瘤最大直径将患者分为 < 2.5 cm、2.5 cm-5.0 cm、 > 5.0 cm三个亚组，三组患者OS（*P*=0.570，图 3A）和PFS（*P*=0.150，图 3B）差异无明显统计学意义。然而，单发肺转移瘤患者的OS（*P*=0.020，图 3C）及PFS（*P*=0.020，图 3D）明显优于多发肺转移瘤患者，差异具有统计学意义。无胸膜转移胸腔积液的患者的OS（*P* < 0.000, 1，图 4A）及PFS（*P* < 0.000, 1，图 4B）明显优于合并胸膜转移胸腔积液的ACC肺转移患者。

**图 3:**
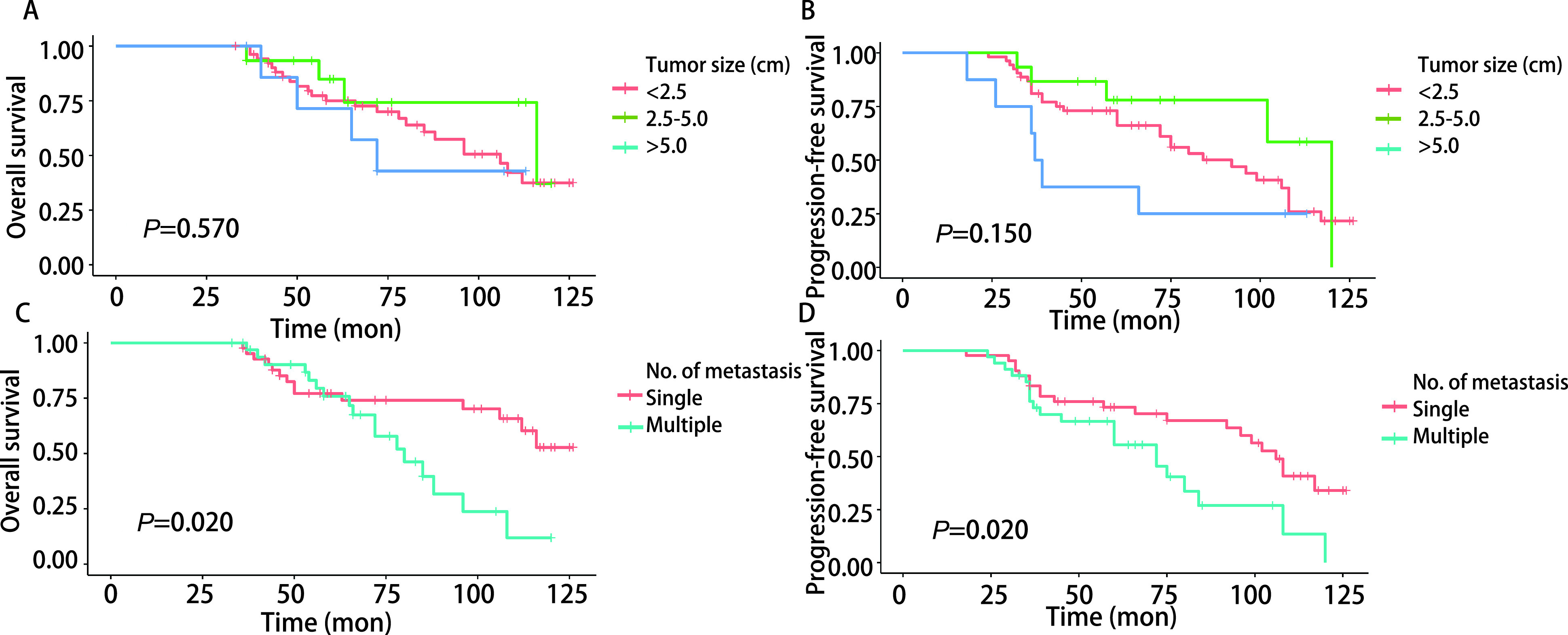
不同肺转移瘤直径及数目亚组患者生存曲线比较。肺转移瘤最大直径 < 2.5 cm、2.5 cm-5.0 cm、 > 5.0 cm三个亚组患者OS（A）和PFS（B）差异无明显统计学意义。单发肺转移瘤患者的OS（C）及PFS（D）明显优于多发肺转移瘤患者，差异具有统计学意义。 Comparison of survival curves of patients with different lung metastasis diameter and number subgroups. There was no significant difference in OS (A) and PFS (B) in the three subgroups of patients with lung metastases with a maximum diameter of < 2.5 cm, 2.5 cm-5.0 cm, and > 5.0 cm. The OS (C) and PFS (D) of patients with single lung metastases were significantly better than those with multiple lung metastases, and the difference was statistically significant.

**图 4:**
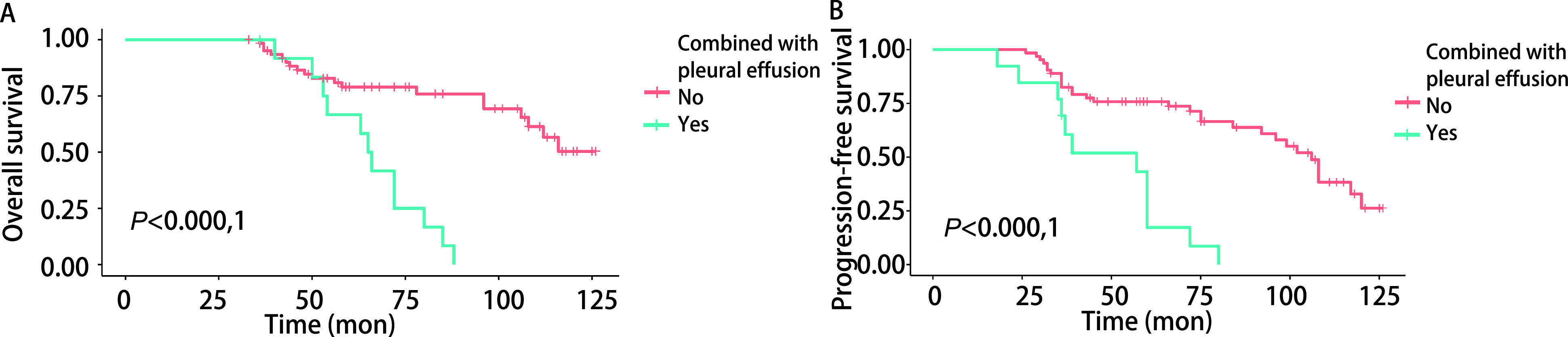
合并胸膜转移胸腔积液ACC患者的生存曲线。无胸膜转移胸腔积液的患者的OS（A）及PFS（B）明显优于合并胸膜转移胸腔积液的患者。 Survival curve of ACC patients with pleural metastasis and pleural effusion. The OS (A) and PFS (B) of patients without pleural effusion were significantly better than those with pleural effusion.

## 3 讨论

头颈部ACC是一种少见的恶性肿瘤，通常被认为是低度恶性肿瘤，其特点是生长缓慢，数年后有复发的趋势^[5, 11]^。有报道称，头颈部ACC原发肿瘤无局部复发的患者也可发生远处转移，疾病进展时会转移至肺、肝、骨等部位，远处转移率为20%-55%^[7, 11]^，其中最常见的转移部位是肺，在发生转移的病例中，肺转移的比例为67.0%-92.9%^[3]^。ACC肺转移通常进展缓慢，并且在很长时间后才出现症状，肺功能可长期维持，与其他器官的远处转移行为不同^[11]^。van Weert等^[13]^报道，肺转移患者的平均OS为32个月，而其他地方的转移性疾病的平均OS为20个月；Gao等^[14]^报道，肺转移患者的中位OS为44个月，而不同转移部位的患者的中位OS为8个月（综合所有其他转移部位）。正因如此，有学者提出，是否将ACC肺转移作为单独的疾病进行治疗^[1, 14]^。然而，大多数关于ACC的文献都是小规模的研究或病例报道^[2, 5, 15]^，目前还没有针对ACC远处转移的特异性治疗指南，尤其是肺转移。

本研究队列包括了76例头颈部ACC肺转移患者，据我们所知，是迄今为止国内报道的样本量规模最大的研究，我们具体描述了中国人头颈部ACC肺转移队列的临床结果并评估了不同治疗策略的远期预后结局。我们的研究表明，ACC肺转移患者非手术治疗的存活率低于手术切除，手术治疗显示了最好的OS和PFS（图 2C，图 2D），这与以前的研究^[1, 3]^一致。这一结果表明，如果可行的话，手术治疗ACC肺转移患者的效果最好。然而，根据初始治疗策略的亚组分析，手术切除联合放疗/化疗组的5年和10年OS率和PFS率虽然优于放化疗组及辅助支持治疗组的患者，但与单纯手术组比较，未见明显统计学差异。Ito等^[5]^认为，ACC转移灶对化疗并不敏感，这可能是因为早期肿瘤所占比例较大和仅有少部分患者接受根治性化疗，有可能存在不利于化疗治疗的生存偏差。Sharma等^[4]^认为对于切缘阳性（R1切除）的ACC患者，放射治疗可获得相当于切缘阴性（R0切除）患者的长期生存时间，这可能是由于该研究回顾性评估少数患者，且各亚组的随访时间不同，可能存在有利于放疗的偏倚。有研究^[13]^显示，与外科手术相比，根治性放疗后患者的5年生存率仅为12%-17%，预后明显较差，这可能是因为接受放射治疗的患者可能有疾病分期更晚，不适合手术切除，或者患者身体状况不佳，不适合手术。Qin等^[8]^的研究发现，与未经手术治疗的患者相比，辅助放疗延长了OS，这与我们的研究一致。本研究中，21例（27.63%）患者接受了放射治疗，30例（39.47%）患者接受了全身化疗，他们的OS和PFS优于那些没有接受特殊治疗（手术、放化疗）的患者，但比接受手术治疗的患者差（图 1A，图 1B，图 2C，图 2D），这进一步说明手术治疗对于ACC肺转移患者的重要性。

肺是头颈部ACC远处转移的主要部位这一观点已得到多数文献的支持^[1, 3, 7, 12]^，肺远处转移可在最初诊断后的很长一段时间内发生，无症状表现^[1, 7]^。本研究中，42例（55.26%）单发肺转移患者均无明显肺部症状，其中6例患者直到首次手术5年后在常规复查胸部CT时才观察到肺转移。这提示头颈部ACC需要警觉的、长期的随访和胸部CT检查，以便在早期和可切除的阶段发现肺转移，从而进行局部治疗以控制疾病播散和转移。Girelli等^[1]^描述了根治性转移瘤切除术治疗肺转移的有益效果。然而，当ACC肺转移灶合并胸膜转移胸腔积液时，往往提示预后较差。本研究13例ACC患者合并胸膜转移胸腔积液（17.11%），其中8例（10.53%）为单侧胸腔积液，5例（6.58%）为双侧胸腔积液，因无法行转移瘤根治手术，化疗药胸腔热灌注及姑息性化疗是此类患者最常见的治疗方法，5年和10年OS率分别为58.33%和0%；无胸膜转移胸腔积液的患者的5年和10年OS率分别为78.94%和50.35%，生存曲线显示OS（图 4A）及PFS（图 4B）明显优于合并胸膜转移胸腔积液的ACC肺转移患者。

越来越多的证据^[16-19]^表明，肿瘤分期是决定头颈部ACC患者预后的主要因素，但以往大多数文献^[2, 5, 14, 15]^报道只是描述了原发病变的肿瘤分期情况，并没有提到肺部疾病的确切进展程度或患者的表现状况，很难对肺部转移病变进行更平衡的比较，因此，本研究根据IRLM分期系统^[1]^（包含原发肿瘤初次治疗至肺转移发生的时间间隔、肺部病变是否完全切除、转移为单发或多发三个因素）对ACC肺转移病变进行分期，评估不同分期患者接受治疗后的相关结局及预后，使研究更加客观且具有可比性。本研究ACC肺转移低分期（Ⅰ期+Ⅱ期）患者的OS（图 1C）和PFS（图 1D）明显优于高分期（Ⅲ期+Ⅳ期）患者；单发肺转移瘤患者的OS（图 3C）及PFS（图 3D）明显优于多发肺转移瘤患者，差异具有统计学意义。这进一步表明了治疗控制原发肿瘤、完全切除肺部病变和早期处理肺部单发转移与良好的长期疗效相关。

综上所述，尽管头颈部ACC往往需要手术、放化疗或辅助支持治疗，但对于ACC肺转移病变的患者，应考虑对肺转移病变进行特异性治疗。我们的研究显示，接受手术治疗的ACC肺转移患者的远期预后优于其他治疗方法，与更好的OS和PFS相关。对于具备手术条件的ACC肺转移的患者，进行完整手术切除的意义应高于其他治疗方案。据我们所知，本研究是目前国内报道的最大规模的头颈部ACC肺转移的研究，探讨了ACC肺转移患者的有效治疗策略、临床结局和长期预后，为中国人头颈部ACC肺转移的指导治疗和未来的指南制定提供更多的信息。然而，我们的研究因其回顾性设计而受到限制，放疗、化疗和支持治疗方案并不统一，不同剂量的化疗药物、放疗剂量、放疗和化疗持续时间对ACC患者预后的影响尚未讨论，亦不能排除患者和治疗选择的偏见。有些结果可能不具有代表性，进一步的前瞻性研究和更大的系列研究有利于降低基线特征不均衡对研究的影响，以获得更有说服力的结论。
